# White matter hyperintensities and smaller cortical thickness are associated with neuropsychiatric symptoms in neurodegenerative and cerebrovascular diseases

**DOI:** 10.1186/s13195-023-01257-y

**Published:** 2023-06-20

**Authors:** Miracle Ozzoude, Brenda Varriano, Derek Beaton, Joel Ramirez, Sabrina Adamo, Melissa F. Holmes, Christopher J. M. Scott, Fuqiang Gao, Kelly M. Sunderland, Paula McLaughlin, Maged Goubran, Donna Kwan, Angela Roberts, Robert Bartha, Sean Symons, Brian Tan, Richard H. Swartz, Agessandro Abrahao, Gustavo Saposnik, Mario Masellis, Anthony E. Lang, Connie Marras, Lorne Zinman, Christen Shoesmith, Michael Borrie, Corinne E. Fischer, Andrew Frank, Morris Freedman, Manuel Montero-Odasso, Sanjeev Kumar, Stephen Pasternak, Stephen C. Strother, Bruce G. Pollock, Tarek K. Rajji, Dallas Seitz, David F. Tang-Wai, John Turnbull, Dar Dowlatshahi, Ayman Hassan, Leanne Casaubon, Jennifer Mandzia, Demetrios Sahlas, David P. Breen, David Grimes, Mandar Jog, Thomas D. L. Steeves, Stephen R. Arnott, Sandra E. Black, Elizabeth Finger, Jennifer Rabin, ONDRI Investigators, Michael Strong, Peter Kleinstiver, Jane Lawrence-Dewar, Natalie Rashkovan, Susan Bronskil, Julia Fraser, Bill McIlroy, Ben Cornish, Karen Van Ooteghem, Frederico Faria, Yanina Sarquis-Adamson, Alanna Black, Barry Greenberg, Wendy Hatch, Chris Hudson, Elena Leontieva, Ed Margolin, Efrem Mandelcorn, Faryan Tayyari, Sherif Defrawy, Don Brien, Ying Chen, Brian Coe, Doug Munoz, Alisia Southwell, Dennis Bulman, Allison Ann Dilliott, Mahdi Ghani, Rob Hegele, John Robinson, Ekaterina Rogaeva, Sali Farhan, Seyyed Mohammad Hassan Haddad, Nuwan Nanayakkara, Courtney Berezuk, Malcolm Binns, Wendy Lou, Athena Theyers, Abiramy Uthirakumaran, Guangyong (GY) Zou, Sujeevini Sujanthan, Mojdeh Zamyadi, David Munoz, Roger A. Dixon, John Woulfe, Brian Levine, J. B. Orange, Alicia Peltsch, Angela Troyer, Marvin Chum, Maria Carmela Tartaglia

**Affiliations:** 1 Tanz Centre for Research in Neurodegenerative Diseases, University of Toronto, Krembil Discovery Tower, 60 Leonard Avenue, 6th floor 6KD-407, Toronto, ON M5T 2S8 Canada; 2 L.C. Campbell Cognitive Neurology Unit, Sunnybrook Health Sciences Centre, Toronto, ON Canada; 3 Dr. Sandra Black Centre for Brain Resilience and Recovery, Hurvitz Brain Sciences Program, Sunnybrook Research Institute, University of Toronto, Toronto, ON Canada; 4 Department of Psychology, Faculty of Health, York University, Toronto, ON Canada; 5 Central Michigan University College of Medicine, Mount Pleasant, MI USA; 6 Data Science & Advanced Analytic, St. Michael’s Hospital, Toronto, ON Canada; 7 Graduate Department of Psychological Clinical Science, University of Toronto Scarborough, Scarborough, ON Canada; 8 Rotman Research Institute of Baycrest Centre, Toronto, ON Canada; 9 Nova Scotia Health, Halifax, NS Canada; 10 Harquail Centre for Neuromodulation, Hurvitz Brain Sciences Program, Sunnybrook Health Sciences Centre, Toronto, ON Canada; 11 Department of Medical Biophysics, University of Toronto, Toronto, ON Canada; 12 Sunnybrook Research Institute, Sunnybrook Health Sciences Centre, Toronto, ON Canada; 13 Centre for Neuroscience Studies, Queen’s University, Kingston, ON Canada; 14 Queen’s University, Kingston, ON Canada; 15 Roxelyn and Richard Pepper Department of Communication Sciences and Disorders, Northwestern University, Evanston, IL USA; 16 School of Communication Sciences and Disorders, Faculty of Health Sciences, Western University, London, ON Canada; 17 Robarts Research Institute, Western University, London, ON Canada; 18 Department of Medicine, Division of Neurology, University of Toronto, Toronto, ON Canada; 19 Heart & Stroke Foundation Canadian Partnership for Stroke Recovery, Sunnybrook Health Sciences Centre, Toronto, ON Canada; 20 Division of Neurology, Department of Medicine, St. Michael’s Hospital, University of Toronto, Toronto, ON Canada; 21 Li Ka Shing Knowledge Institute, St. Michael’s Hospital, University of Toronto, Toronto, ON Canada; 22 Edmond J Safra Program for Parkinson Disease, Movement Disorder Clinic, Toronto Western Hospital, University Health Network, Toronto, ON Canada; 23 Department of Clinical Neurological Sciences, Western University, London, ON Canada; 24 Schulich School of Medicine and Dentistry, Western University, London, ON Canada; 25 Keenan Research Centre for Biomedical Science, St. Michael’s Hospital, Toronto, ON Canada; 26 Department of Medicine (Neurology), University of Ottawa Brain and Mind Research Institute, Ottawa, ON Canada; 27 Bruyère Research Institute, Ottawa, ON Canada; 28 Division of Neurology, Baycrest Health Sciences, Toronto, ON Canada; 29 Lawsone Health Research Institute, London, ON Canada; 30 Gait and Brain Lab, Parkwood Institute, London, ON Canada; 31 Department of Psychiatry, University of Toronto, Toronto, ON Canada; 32 Adult Neurodevelopment and Geriatric Psychiatry, Centre for Addiction and Mental Health, Toronto, ON Canada; 33 Toronto Dementia Research Alliance, University of Toronto, Toronto, ON Canada; 34 Cumming School of Medicine, University of Calgary, Calgary, AB Canada; 35 Memory Clinic, Toronto Western Hospital, University Health Network, Toronto, ON Canada; 36 Faculty of Health Sciences, McMaster University, Hamilton, ON Canada; 37 Department of Medicine, McMaster University, Hamilton, ON Canada; 38 Thunder Bay Regional Health Research Institute, Thunder Bay, ON Canada; 39 St. Joseph’s Healthcare Centre, London, ON Canada; 40 Centre for Clinical Brain Sciences, University of Edinburgh, Edinburgh, UK; 41 Anne Rowling Regenerative Neurology Clinic, University of Edinburgh, Edinburgh, UK; 42 Usher Institute of Population Health Sciences and Informatics, University of Edinburgh, Edinburgh, UK; 43 London Health Sciences Centre, London, ON Canada; 44 Rehabilitation Sciences Institute, University of Toronto, Toronto, ON Canada

**Keywords:** White matter hyperintensities, Cortical thickness, Neuropsychiatric symptoms, Neurodegenerative disease, Cerebrovascular disease

## Abstract

**Background:**

Neuropsychiatric symptoms (NPS) are a core feature of most neurodegenerative and cerebrovascular diseases. White matter hyperintensities and brain atrophy have been implicated in NPS. We aimed to investigate the relative contribution of white matter hyperintensities and cortical thickness to NPS in participants across neurodegenerative and cerebrovascular diseases.

**Methods:**

Five hundred thirteen participants with one of these conditions, i.e. Alzheimer’s Disease/Mild Cognitive Impairment, Amyotrophic Lateral Sclerosis, Frontotemporal Dementia, Parkinson’s Disease, or Cerebrovascular Disease, were included in the study. NPS were assessed using the Neuropsychiatric Inventory – Questionnaire and grouped into hyperactivity, psychotic, affective, and apathy subsyndromes. White matter hyperintensities were quantified using a semi-automatic segmentation technique and FreeSurfer cortical thickness was used to measure regional grey matter loss.

**Results:**

Although NPS were frequent across the five disease groups, participants with frontotemporal dementia had the highest frequency of hyperactivity, apathy, and affective subsyndromes compared to other groups, whilst psychotic subsyndrome was high in both frontotemporal dementia and Parkinson’s disease. Results from univariate and multivariate results showed that various predictors were associated with neuropsychiatric subsyndromes, especially cortical thickness in the inferior frontal, cingulate, and insula regions, sex(female), global cognition, and basal ganglia-thalamus white matter hyperintensities.

**Conclusions:**

In participants with neurodegenerative and cerebrovascular diseases, our results suggest that smaller cortical thickness and white matter hyperintensity burden in several cortical-subcortical structures may contribute to the development of NPS. Further studies investigating the mechanisms that determine the progression of NPS in various neurodegenerative and cerebrovascular diseases are needed.

## Background

Neuropsychiatric symptoms (NPS) (such as depression, anxiety, apathy, psychosis, and disinhibition) are commonly reported in neurodegenerative and cerebrovascular diseases [1]. Their high frequency and increased severity are associated with higher patient distress, increased caregiver burden, and higher rates of institutionalised care [2, 3]. Moreover, the frequency of NPS varies across the various neurodegenerative and cerebrovascular disease. Affective symptoms like anxiety and depression are more prevalent in Alzheimer’s disease (AD) and vascular dementia (VaD) [4–7]. Apathy is most commonly reported in AD and frontotemporal dementia (FTD), and associated with functional impairment and disease progression [8, 9]. But the pattern of apathy presentation differs such that AD-related apathy is indicative of depression, cognitive dysfunction, and conversion from amnestic mild cognitive impairment (aMCI) to AD [10, 11], whilst FTD-related apathy is associated with measures of social cognition and executive dysfunction [11–13]. Moreover, the apathy symptoms observed in FTD have also been reported in ALS with behavioural variant FTD (bvFTD) [14, 15]. In Parkinson’s disease (PD), depression and anxiety are also present in addition to apathy, fatigue, sleep disturbances, and psychosis [16, 17]. Since the manifestation of NPS likely represents brain abnormalities and may reflect progression of disease, it is important to recognise the neural basis of NPS in neurodegenerative and cerebrovascular diseases.

Regional brain changes have been implicated in NPS in neurodegenerative and cerebrovascular diseases [5, 12, 15, 18–23]. Symptoms of apathy, anxiety, and depression reported in aMCI have been linked to smaller cortical thickness and volume in the frontal, temporal, and parietal regions [5, 19, 21]. In PD, lower frontal lobe volume was related to affective, psychotic, and apathy symptoms [22], whilst a smaller cortical thickness and volume of the frontotemporal, insular, and limbic regions was related to apathy and disinhibition in FTD and ALS [12, 15, 23]. In cerebrovascular disease (CVD), appetite/eating behaviour, depression, and apathy has been associated with smaller hippocampal, middle, and posterior cingulate volumes [24–26]. Although these studies suggest that NPS, particularly affective, apathy, and psychotic symptoms are most frequently associated with grey matter alterations in the fronto-subcortical circuitries, white matter lesions such as white matter hyperintensities (WMH) have also been implicated in NPS [27–32].

WMH have traditionally been attributed to either cerebrovascular disease [33], ageing [34], or neuroinflammatory processes [35]. In most neurodegenerative diseases, they are attributed to small vessel disease (SVD) [33]. However, these assumptions are being questioned as there is increasing evidence that non-vascular pathology such as tau-mediated secondary demyelination or microglial dysfunction may also contribute to WMH in neurodegenerative diseases [36]. In the context of presumed vascular origin, WMH in the frontal, parieto-occipital, and basal ganglia areas have been related to psychotic symptoms in AD [27]. Furthermore, greater WMH load (particularly in the frontal lobe) has been associated with greater delusions, hallucinations, anxiety, apathy, and depression in both AD and VaD [28–32, 37], as well as severe apathy and night time behaviour in FTD [37], and depression in PD with dementia [38].

Whilst changes in brain thickness, volume, and WMH burden have been associated with NPS, not many studies have investigated their contributions to NPS across multiple neurodegenerative and cerebrovascular diseases. This limitation may be partly due to the lack of transdiagnostic datasets, as previous research have focused on analyses within a single disease [39] or multiple diseases, mainly consisting of VaD, AD/MCI, and mixed dementia [31, 40], occasionally PD and FTD [30, 37], and none on ALS. Thus, the aims of the present study were to compare the frequency of NPS across multiple neurodegenerative and cerebrovascular diseases and to determine its relationship with WMH burden and cortical thickness across all cohorts. We hypothesised that all cohorts would display high frequency of NPS, particularly in participants with FTD and it will be associated with both WMH burden and a smaller focal cortical thickness.

## Methods

### Participants and study design

Study participants were enrolled as part of Ontario Neurodegenerative Disease Research Initiative (ONDRI), a multi-centre, multiple assessment, longitudinally observational study conducted in nine tertiary care academic medical centres in Ontario, Canada. Detailed inclusion and exclusion criteria for each diagnostic cohort (dx) are reported elsewhere [41, 42]. Briefly, AD/MCI participants met National Institute on Aging Alzheimer’s Association criteria for probable or possible AD, or aMCI [43, 44]; ALS participants met El Escorial World Federation of Neurology diagnostic criteria for possible, probable, or definite familial or sporadic ALS [45]; the latest criteria were used for possible or probable bvFTD [46], for agrammatic/non-fluent and semantic variants of primary progressive aphasia (nfvPPA and svPPA) [47] and possible or probable progressive supranuclear palsy (PSP) and corticobasal syndrome (CBS) [48]; PD participants met criteria for idiopathic PD defined by the United Kingdom’s Parkinson’s Disease Society Brain Bank clinical diagnostic criteria [49]; and CVD participants had experienced a mild or moderate ischemic stroke event (documented on MRI or CT) 3 or more months prior to enrolment in compliance with the National Institute of Neurological Disorders and Stroke-Canadian Stroke Network vascular cognitive impairment harmonisation standards [50]. The study was approved by each participating institution’s Research Ethics Board and performed in accordance with the Declaration of Helsinki. All participants provided informed consent and subsequently underwent clinical evaluation and MRI, in addition to the other assessments as part of the full ONDRI protocol described elsewhere [41]. The current project only used data from the baseline evaluation.

## Measures

### Neuropsychiatric symptoms (NPS) assessment

The Neuropsychiatric Inventory-Questionnaire (NPI-Q) was used to assess NPS observed in dementia [51]. Specifically, the study partners completed a questionnaire, where they indicated the presence and severity (mild, moderate, and severe) of 12 common NPS. This questionnaire also measured the level of distress (on a 5-point scale) the NPS caused the study partner. A total NPI-Q severity score was the sum of all the individual symptom severity scores and the total NPI-Q study partner distress score was the sum of all the individual symptom study partner distress scores. For the current study, we classified the symptoms into four neuropsychiatric subsyndrome groups in accordance with the European Alzheimer’s Disease Consortium [52], and the score for each subsyndrome was the sum of all the symptom severity scores in the subsyndrome: hyperactivity subsyndrome (agitation/aggression, euphoria/elation, irritability/lability, disinhibition, and aberrant motor behaviour); psychotic subsyndrome (hallucinations, delusions, and night time behaviours); affective subsyndrome (depression/dysphoria and anxiety); and apathy subsyndrome (apathy/indifference and appetite/eating).

### Global cognitive and functional assessments

Global cognitive function was evaluated using the Montreal Cognitive Assessment (MoCA) on all participants [53] for which the total score was adjusted for educational attainment, and they were rated on instrumental activity of daily living (iADLs) and activity of daily living (ADLs) by their study partners [54].

### Vascular risk factors

Participants were considered to have vascular risk factors if they reported to have received a diagnosis of hypertension, diabetes, and/or high cholesterol during medical history interview in addition to smoking history. Furthermore, we created a total measure of vascular risk factors burden per participant by counting the occurrences where they indicated a diagnosis of hypertension, diabetes, and/or high cholesterol, and having ever smoked for 3 or more months.

### MRI acquisition

MRI scans were acquired using 3 Tesla MRI systems. MRI protocols details are published elsewhere [55, 56] and harmonised with the Canadian Dementia Imaging Protocol (CDIP) [57]. Briefly, the structural MRI used in this specific analysis of ONDRI data included the following sequences: high-resolution three-dimensional T1-weighted, interleaved proton density, T2-weighted, and T2 fluid-attenuated inversion recovery.

## Image processing

### White matter hyperintensity estimation

A detailed description of ONDRI structural processing pipeline methods has been described elsewhere [56]. Briefly, ONDRI’s neuroimaging platform used previously published and validated methods [58–64] and outputs were further subjected to comprehensive quality control measures from ONDRI’s neuroinformatics platform [65]. The final output of the neuroimaging pipeline produced a skull-stripped brain mask with segmented voxels comprising normal appearing white matter, normal appearing grey matter, ventricular and sulcal cerebrospinal fluid, deep and periventricular lacunes, perivascular spaces, cortico-subcortical stroke lesion, periventricular WMH (pWMH), and deep WMH (dWMH). The 10 tissue classes were further combined with ONDRI’s 28 regional parcellation to create 280 distinct brain regions [56].

For the purpose of this study, we combined both pWMH and dWMH volumes. This was derived by extracting brain parcellations that intersected with WMH segmentation and adding them to create 5 regional WMH volumes: frontal, parietal, temporal, occipital, and basal ganglia/thalamus (BGT)**.** Each regional WMH volume was brain volume corrected using supratentorial total intracranial volume (ST-TIV) and log transformed + small constant to achieve normal distribution:

- Corrected and transformed regional WMH volume = log((*x* / STTIV) + 0.0001); where *x* = uncorrected and untransformed regional WMH volume.

### Cortical thickness estimation

All scans were processed using the stable version of FreeSurfer (FS) (Linux FSv6.0). Details of FreeSurfer pipeline have been previously described [66, 67]. Briefly, the standard reconstruction steps included skull stripping, WM segmentation, intensity normalisation, surface reconstruction, subcortical segmentation, cortical parcellation, and thickness. A modified FreeSurfer pipeline was used that incorporated ONDRI’s skull stripped and lesion masks to decrease overall failure rates in participants with significant atrophy and SVD [68].

Cortical thickness was measured as the distance between the GM and WM boundaries (WM surface) to GM and CSF boundaries (pial surface) on the cortex in each hemisphere. We extracted the 68 cortical thickness regions from the Desikan-Killany atlas for further regression analyses [69].

## Statistical analyses

Statistical analyses were conducted using R (v 3.4.1) and figures generated using ggplot2 package [70]. One-way ANOVA was used to determine group differences on age, education, MoCA score, ADLs, iADLs, NPI-Q total severity, and NPI-Q caregiver distress. Chi-square test was performed to look for group differences in sex, history of vascular risk factors, and frequencies of NPS across groups. One-way MANOVA was conducted to determine group differences on hyperactivity, psychotic, affective, and apathy subsyndromes. Sex differences in frequencies of NPS were performed using chi-square or Fisher’s exact tests where appropriate. Group differences on ST-TIV adjusted log transformed regional WMH volumes was analysed using one-way MANCOVA, whilst controlling for age. Bonferroni post hoc correction was used where applicable. We ran a linear regression to examine the association between total vascular risk factors burden and log transformed ST-TIV corrected total WMH load, adjusted for age and sex.

### Elastic net models and partial least square correlation

The two approaches that we used to determine the relationships between neuropsychiatric subsyndromes, cortical thickness regions, and lobar WMH volumes have been described in details elsewhere [71]. Firstly, we employed a univariate approach with elastic net (LASSO + ridge penalised regression) which is a sparse (LASSO) and penalised (ridge) procedure that suppresses coefficients to zero and helps identify the best subset of explanatory variables for a dependent variable [72, 73]. Each elastic net model consisted of neuropsychiatric subsyndrome ~ sex + age + MoCA + 68 cortical thickness regions + 10 lobar WMH. Alpha was set to equals 1 which was the elastic net penalty parameter for LASSO and we used *glmnet’s* internal cross-validation to search over the lambda parameter (ridge). Using a repeated train-test procedure, 75% of the data was used for internal cross-validation to identify the lambda parameter with k-folds equals 10, whilst the remaining 25% were used to test the model and report the lambda values with the mean square error (MSE). The above steps were repeated 500 times to construct a consensus of variables with the lowest MSE from the test step. We identified all models from the 500 repeats where a lambda value corresponded to the lowest MSE approximately 5% of the time. That is, models corresponding to lambda values that appeared approximately 25/500 times were retained, and those variables saved. We preserved the sex-by-dx distribution of the entire sample for the repeated splits.

Secondly, a multivariate approach with partial least square correlation (PLSc) was used to model the relationship between all four neuropsychiatric subsyndromes and the independent variables (sex, age, MoCA, cortical thickness regions, and lobar WMH volumes). Two resampling methods were used to help identify which components to interpret (permutation) [74–76], and to identify which variables were the most stable contributors to the components (bootstrap) [74, 77, 78]. We also preserved the sex-by-dx distribution of the entire sample for resampling.

## Results

### Participant demographic and clinical characteristics

A total of 513 participants (AD/MCI (*N* = 126), ALS (*N* = 40), FTD (*N* = 52), PD (*N* = 140), and CVD (*N* = 155)) with available baseline MRIs were included in this analysis. In the FTD group, 21 (40.4%) were diagnosed with bvFTD, 8 (15.4%) were diagnosed with nfvPPA, 4 (7.7%) were diagnosed with svPPA, 16 (30.8%) were diagnosed with PSP-Richardson syndrome, and 3 (5.8%) were diagnosed with CBS. Participants’ demographic and clinical characteristics are displayed in Table 1. All groups differed in terms of age, education, sex, MoCA, ADLs, iADLs, hypertension, and high cholesterol.

**Table 1:** Demographic, clinical, and neuroimaging characteristics across diagnostic groups

	**AD/MCI** **(*N* = 126)** **Mean (SD)**	**ALS** **(*N* = 40)** **Mean (SD)**	**FTD** **(*N* = 52)** **Mean (SD)**	**PD** **(*N* = 140)** **Mean (SD)**	**CVD** **(*N* = 155)** **Mean (SD)**	**Effect size** η^2^/V	**F/***χ*^**2**^**, *p-*value**
Age (years)	71.03 (8.16)	61.98 (8.74)	67.81 (7.12)	67.94 (6.34)	69.35 (7.36)	η^2^ = 0.09	*F*_*(*4,508)_ = 12.18, *p* < 0.001^a^
Sex (F:M) (% F)	57:69 (45.2)	16:24 (40.0)	19:33 (36.5)	31:109 (22.1)	49:106 (31.6)	V = 0.18	*χ* ^2^ (4) = 17.11, *p* = 0.002
Education (years)	15.23 (3.08)	13.83 (2.88)	13.89 (2.73)	15.49 (2.73)	14.69 (2.88)	η^2^ = 0.04	*F*_*(*4,508)_ = 5.09, *p* = 0.001^b^
MoCA total score	22.67 (2.99)	25.46 (2.83)	21.48 (3.96)	25.84 (2.57)	25.29 (2.99)	η^2^ = 0.22	*F*_*(*4,507)_ = 12.18, *p* < 0.001^c^
ADLs	98.15 (4.59)	87.50 (13.95)	87.58 (15.65)	96.56 (7.34)	98.32 (5.42)	η^2^ = 0.19	*F*_*(*4,483)_ = 27.92, *p* < 0.001^d^
iADLs	85.28 (17.29)	78.27 (21.67)	60.99 (27.70)	89.73 (14.06)	91.13 (14.21)	η^2^ = 0.21	*F*_*(*4,474)_ = 32.05, *p* < 0.001^e^
***Vascular risk*** ***factors, n (% yes)***							
Hypertension	34 (64.2)	10 (71.4)	19 (70.4)	47 (69.1)	113 (83.7)	V = 0.19	*χ* ^2^ (4) = 10.46, *p* = 0.036
Diabetes	25 (34.2)	2 (10.5)	8 (27.6)	13 (19.1)	34 (26.2)	V = 0.14	*χ* ^2^ (4) = 6.69, *p* = 0.159
High cholesterol	58 (79.5)	12 (63.2)	27 (93.1)	57 (83.8)	121 (93.1)	V = 0.24	*χ* ^2^ (4) = 17.94, *p* = 0.001
Smoking	67 (53.2)	22 (55.0)	28 (53.8)	58 (41.4)	84 (54.2)	V = 0.11	*χ* ^2^ (4) = 6.37, *p* = 0.173
***NPI-Q***	**Mean (SD)**	**Mean (SD)**	**Mean (SD)**	**Mean (SD)**	**Mean (SD)**		
NPI-Q total severity score	3.67 (3.96)	3.08 (3.68)	8.08 (6.24)	3.51 (3.92)	3.16 (3.88)	η^2^ = 0.11	*F*_*(*4,469)_ = 14.00, *p* < 0.001^f^
NPI-Q caregiver distress score	4.12 (5.01)	3.74 (5.29)	9.00 (9.18)	4.05 (5.69)	3.55 (5.34)	η^2^ = 0.07	*F*_*(*4,461)_ = 8.28, *p* < 0.001^g^
***Neuropsychiatric subsyndromes***	**Mean (SE)**	**Mean (SE)**	**Mean (SE)**	**Mean (SE)**	**Mean (SE)**		
Affective	0.81 (0.11)	0.70 (0.18)	1.35 (0.16)	0.86 (0.10)	0.57 (0.10)	η^2^ = 0.04	*F*_*(*4,486)_ = 4.46, *p* = 0.002 h
Apathy	0.86 (0.11)	0.76 (0.19)	2.14 (0.17)	0.76 (0.11)	0.68 (0.10)	η^2^ = 0.11	*F*_*(*4,486)_ = 14.53, *p* < 0.001^i^
Hyperactivity	1.49 (0.18)	0.98 (0.31)	3.41 (0.27)	0.86 (0.17)	1.27 (0.16)	η^2^ = 0.12	*F*_*(*4,486)_ = 17.09, *p* < 0.001^j^
Psychosis	0.48 (0.10)	0.43 (0.16)	1.39 (0.15)	1.01 (0.09)	0.60 (0.09)	η^2^ = 0.08	*F*_*(*4,486)_ = 10.45, *p* < 0.001^k^
***Regional WMH (mm***^***3***^***)*†**	**Adjusted mean (SE)**	**Adjusted mean (SE)**	**Adjusted mean (SE)**	**Adjusted mean (SE)**	**Adjusted mean (SE)**		
Frontal	1508.59 (296.13)	1792.83 (535.23)	1797.59 (455.46)	1957.33 (277.74)	3744.89 (263.99)	η^2^ = 0.08	*F*_*(*4,507)_ = 10.37, *p* < 0.001^l^
Parietal	1090.54 (335.25)	1942.71 (605.93)	1401.15 (515.62)	1715.61 (314.43)	3748.51 (298.86)	η^2^ = 0.09	*F*_*(*4,507)_ = 13.19, *p* < 0.001^m^
Occipital	655.97 (74.45)	750.73 (134.57)	599.54 (114.51)	759.25 (69.83)	900.43 (66.37)	η^2^ = 0.02	*F*_*(*4,507)_ = 2.19, *p* = 0.069
Temporal	525.24 (94.64)	644.06 (171.05)	599.86 (145.56)	664.23 (88.76)	1245.17 (84.37)	η^2^ = 0.07	*F*_*(*4,507)_ = 9.57, *p* < 0.001^n^
BGT	82.59 (24.51)	69.62 (44.30)	118.05 (37.69)	181.31 (23.99)	267.43 (21.85)	η^2^ = 0.09	*F*_*(*4,507)_ = 12.55, *p* < 0.001 ^o^

η^2^** = **Partial Eta Squared V = Cramer’s V

*AD* Alzheimer’s disease, *ADLs* Activities of daily living, *ALS* Amyotrophic lateral sclerosis, *BGT* Basal ganglia/thalamus, *CVD* Cerebrovascular disease; *FTD* Frontotemporal disease, *iADLs* instrumental activities of daily living, *MCI* Mild cognitive impairment, *MoCA* Montreal Cognitive Assessment, *NPI-Q* Neuropsychiatric Symptoms Inventory Questionnaire, *PD* Parkinson’s disease

^a^ ALS < AD/MCI ( *p* < 0.001), FTD ( *p* = 0.002), PD (*p* < 0.001), and CVD ( *p* < 0.001); PD < AD/MCI ( *p* = 0.007)

^b^ FTD < AD/MCI ( *p* = 0.047) and PD ( *p* = 0.007); ALS < PD ( *p* = 0.014)

^c^ AD/MCI < ALS, PD, and CVD ( *p* < 0.001); FTD < ALS, PD and CVD ( *p* < 0.001).

^d^ ALS < AD/MCI, PD, and CVD ( *p* < 0.001); FTD < AD/MCI, PD, and CVD ( *p* < 0.001)

^e^ ALS < PD ( *p* = 0.003) and CVD ( *p* < 0.001); FTD < AD/MCI, ALS, PD, and CVD ( *p* < 0.001)

^f^ FTD > AD/MCI, ALS, PD, and CVD ( *p* < 0.001)

^g^ FTD > AD/MCI, PD, and CVD ( *p* < 0.001), FTD > ALS ( *p* = 0.001)

^h^ FTD > CVD (*p* < 0.001)

^i^ FTD > AD/MCI, ALS, PD, and CVD (*p* < 0.001)

^j^ FTD > AD/MCI, ALS, PD, and CVD (*p* < 0.001)

^k^ FTD > AD/MCI, ALS, and CVD (*p* < 0.001); PD > AD/MCI (*p* = 0.001), ALS (*p* = 0.020), and CVD (*p* = 0.011)

^l^ CVD > AD/MCI and PD ( *p* < 0.001); CVD > ALS (*p* = 0.004)

^m^ CVD > AD/MCI, ALS, and PD ( *p* < 0.001); CVD > FTD ( *p* = 0.001)

^n^ CVD > AD/MCI ( *p* < 0.001), ALS ( *p* = 0.002), FTD ( *p* = 0.013), and PD ( *p* = 0.005)

^o^ CVD > AD/MCI ( *p* < 0.001), ALS ( *p* = 0.003), and FTD ( *p* = 0.023); PD > AD/MCI ( *p* < 0.001)

**† **Controlled for age

Results after Bonferroni post hoc correction showed that there were significant differences across all five dx groups on four lobar WMH volumes adjusting for age, with the CVD group showing the highest lobar WMH volumes (Table 1). There was a significant association between total vascular risk factors and WMH load (*β* = 0.176; *p* < 0.001; CI = 0.094–0.244), i.e. having a larger number of vascular risk factors burden was related to increased WMH load after adjusting for age and sex.

### NPS across dx groups

**Fig. 1:**
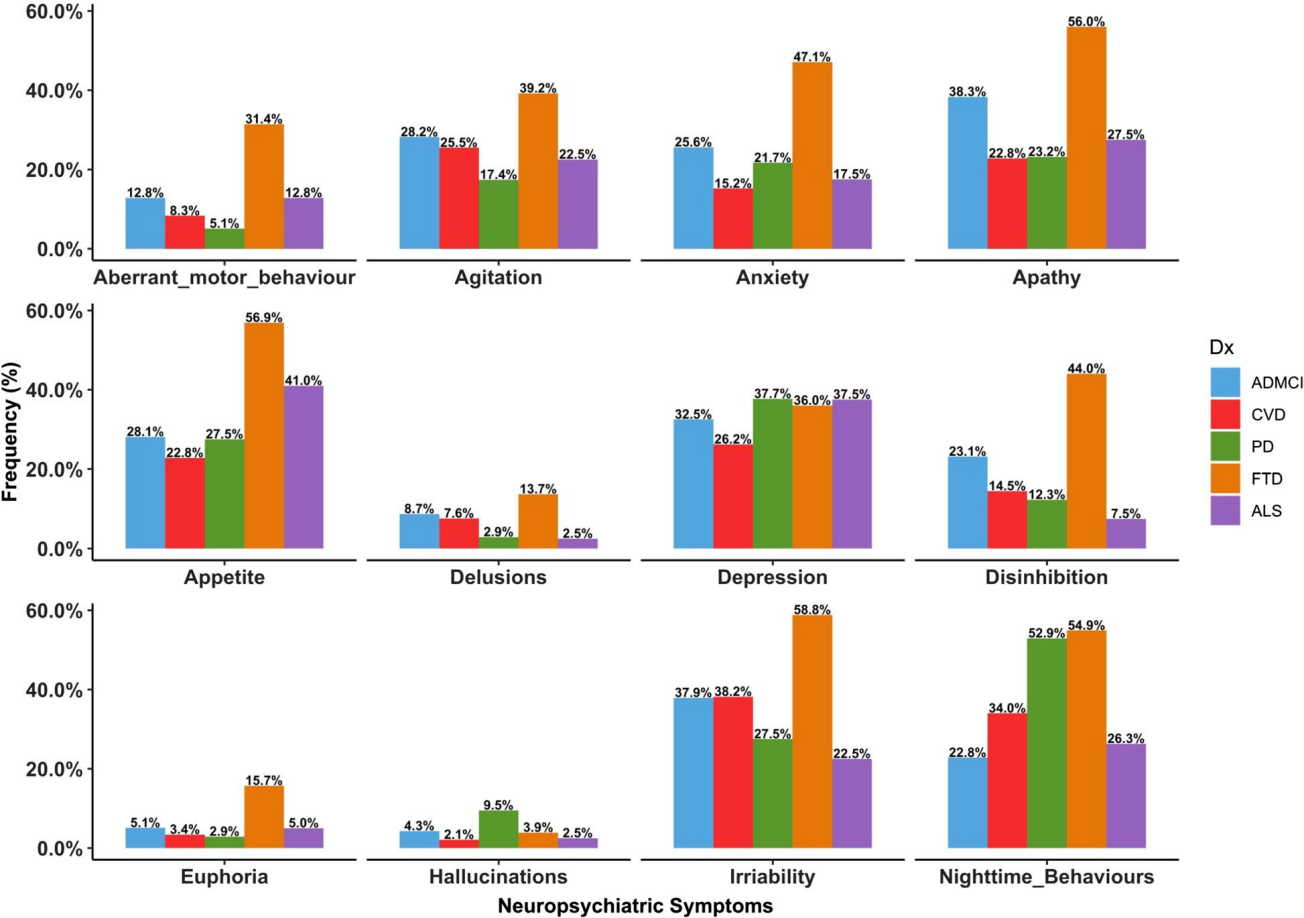
Frequency of neuropsychiatric symptoms (NPS) in various neurodegenerative and cerebrovascular diseases. *Notes*: AD, Alzheimer’s disease; ALS, amyotrophic lateral sclerosis; CVD, cerebrovascular disease; FTD, frontotemporal disease; MCI, mild cognitive impairment; PD, Parkinson’s disease

Although NPS were common across the five disease groups, participants with FTD had the highest frequencies (Table 2; Fig. 1). Agitation, anxiety, apathy, appetite, disinhibition, euphoria, irritability, aberrant motor behaviour, and nighttime behaviours were significantly different across the groups (Table 2; Fig. 1). Depressive symptoms were the most common symptom across all the groups (Table 2). Table 3 shows the group comparison results for significant NPS.

**Table 2:** Frequency of NPS across groups

**NPS,* n* (% yes)**	**AD/MCI**	**ALS**	**FTD**	**PD**	**CVD**	*χ* ^2^***, p*-value**
Delusions	10 (8.7)	1 (2.5)	7 (13.7)	4 (2.9)	11 (7.6)	*χ* ^2^ (4) = 9.12,* p* = 0.058^**‡**^
Hallucinations	5 (4.3)	1 (2.5)	2 (3.9)	13 (9.5)	3 (2.1)	*χ* ^2^ (4) = 9.33,* p* = 0.053^**‡**^
Agitation/aggression	33 ( 28.2)	9 (22.5)	20 (39.2)	24 (17.4)	37 (25.5)	*χ* ^2^ (4) = 10.54,* p* = 0.032*
Depression/dysphoria	38 (32.5)	15 (37.5)	18 (36.0)	52 (37.7)	38 (26.2)	*χ* ^2^ (4) = 4.98,* p* = 0.289
Anxiety	30 (25.6)	7 (17.5)	24 (47.1)	30 (21.7)	22 (15.2)	*χ* ^2^ (4) = 22.94, *p* < 0.001***
Euphoria/elation	6 (5.1)	2 (5.0)	8 (15.7)	4 (2.9)	5 (3.4)	*χ* ^2^ (4) = 14.03, *p* = 0.007**
Apathy/indifference	44 (38.3)	11 (27.5)	28 (56.0)	32 (23.2)	33 (22.8)	*χ* ^2^ (4) = 26.43, *p* < 0.001***
Disinhibition	27 (23.1)	3 (7.5)	22 (44.0)	17 (12.3)	21 (14.5)	*χ* ^2^ (4) = 31.62, *p* < 0.001***
Irritability/lability	44 (37.9)	9 (22.5)	30 (58.8)	38 (27.5)	55 (38.2)	*χ* ^2^ (4) = 19.48, *p* < 0.001***
Aberrant motor behaviour	15 (12.8)	5 (12.8)	16 (31.4)	7 (5.1)	12 (8.3)	*χ* ^2^ (4) = 27.68, *p* < 0.001***
Appetite/eating abnormalities	32 (28.1)	16 (41.0)	29 (56.9)	38 (27.5)	33 (22.8)	*χ* ^2^ (4) = 23.79, *p* < 0.001***
Night time behaviour	26 (22.8)	10 (26.3)	28 (54.9)	73 (52.9)	49 (34.0)	*χ* ^2^ (4) = 33.38, *p* < 0.001***

*AD* Alzheimer’s disease, *ALS* Amyotrophic lateral sclerosis, *CVD* Cerebrovascular disease, *FTD* Frontotemporal disease, *MCI* Mild cognitive impairment, *NPS* Neuropsychiatric symptoms, *PD* Parkinson’s disease

^**‡**^Trending

^***^p < 0.001

^**^p < 0.01

^*^p < 0.05

**Table 3:** Group comparison results for significant NPS

NPS	*P-*value
**Agitation**	
FTD (39.2%) > PD (17.4%)	0.003
**Anxiety**	
FTD (47.1%) > PD (21.7%)	0.001
FTD (47.1%) > ALS (17.5%)	0.004
FTD (47.1%) > CVD (15.2%)	< 0.001
AD/MCI (25.6%) > CVD (15.2%)	0.043**†**
**Apathy**	
FTD (56.0%) > PD (23.2%)	< 0.001
FTD (56.0%) > ALS (27.5%)	0.001
FTD (56.0%) > CVD (22.8%)	< 0.001
AD/MCI (38.3%) > PD (23.2%)	0.013**†**
AD/MCI (38.3%) > CVD (22.8%)	0.009**†**
**Appetite**	
FTD (56.9%) > AD/MCI (28.1%)	< 0.001
FTD (56.9%) > PD (27.5%)	< 0.001
FTD (56.9%) > CVD (22.8%)	< 0.001
ALS (41.0%) > CVD (22.8%)	0.026**†**
**Disinhibition**	
FTD (44.0%) > CVD (14.5%)	< 0.001
FTD (44.0%) > PD (12.3%)	< 0.001
FTD (44.0%) > ALS (7.5%)	< 0.001
AD/MCI (23.1%) > PD (12.3%)	0.030**†**
AD/MCI (23.1%) > ALS (7.5%)	0.036**†**
**Euphoria**	
FTD (15.7%) > PD (2.9%)	< 0.001
**Irritability**	
FTD (58.8%) > PD (27.5%)	< 0.001
FTD (58.8%) > ALS (22.5%)	< 0.001
**Aberrant motor behaviour**	
FTD (31.4%) > CVD (8.3%)	< 0.001
FTD (31.4%) > PD (5.1%)	< 0.001
AD/MCI (12.8%) > PD (5.1%)	0.042**†**
**Night time behaviours**	
FTD (54.9%) > AD/MCI (22.8%)	< 0.001
PD (52.9%) > ADMCI (22.8%)	< 0.001

Comparisons calculated according to Fisher’s exact test

*AD* Alzheimer’s disease, *ALS* Amyotrophic lateral sclerosis, *CVD* Cerebrovascular disease, *FTD* Frontotemporal disease, *MCI* Mild cognitive impairment, *NPS* Neuropsychiatric symptoms, *PD* Parkinson’s disease

^**†**^ Did not survive correction for multiple comparisons (0.05/10 = 0.005)

**Fig. 2:**
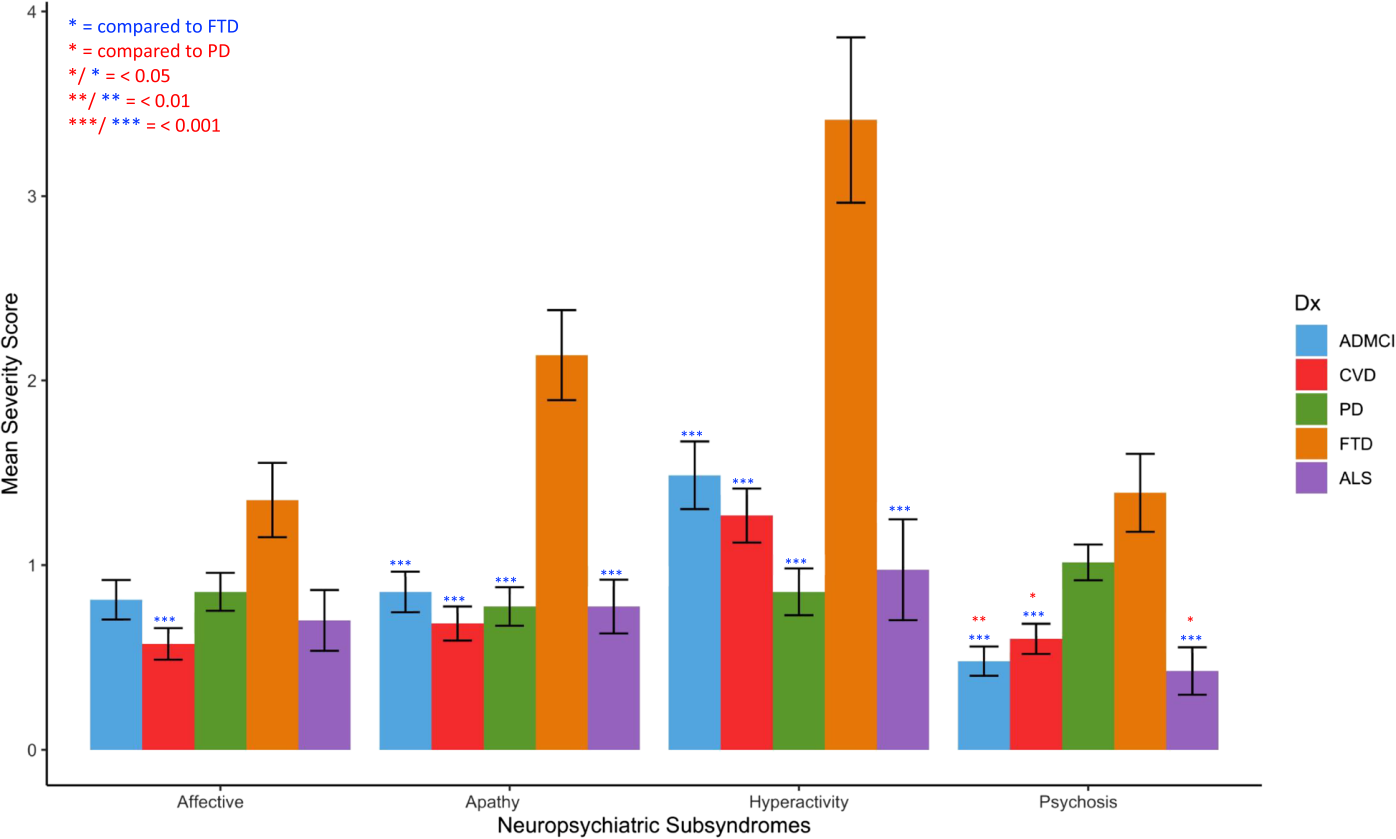
Cluster bar graph showing group differences on neuropsychiatric subsyndromes. *Notes*: AD, Alzheimer’s disease; ALS, amyotrophic lateral sclerosis; CVD, cerebrovascular disease; FTD, frontotemporal disease; MCI, mild cognitive impairment; PD, Parkinson’s disease

Comparing neuropsychiatric subsyndromes across groups showed that hyperactivity, apathy, and affective subsyndromes were highest in FTD compared to other groups, whilst psychotic subsyndrome was high in both FTD and PD (Table 1; Fig. 2).

### Sex differences in frequencies of NPS

**Fig. 3:**
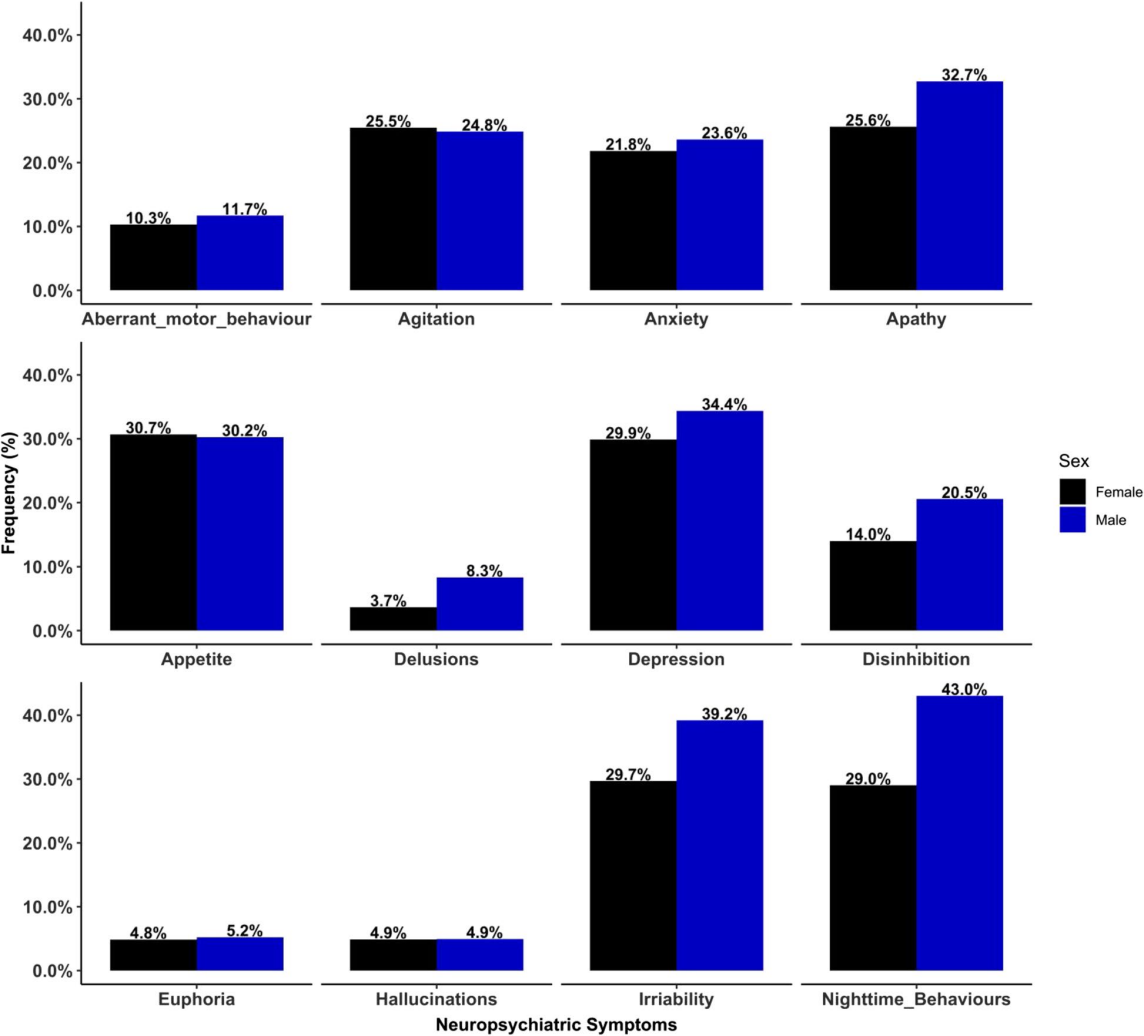
Frequency of neuropsychiatric symptoms (NPS) by sex across the entire sample

Figure 3 shows the sex comparisons of the frequency of individual NPS across the entire sample. Overall, a significantly higher number of males exhibited irritability (39.2% vs 29.7%, *p* = 0.038, *χ*^2^ (1) = 4.28) and nighttime behaviours (43.0% vs 29.0%, *p* = 0.003, *χ*^2^ (1) = 8.97) than females, respectively. The frequency of other NPS was not significant between sexes.

In participants with AD/MCI, depression was significantly higher in females (42.3% vs 24.6%, *p* = 0.042, *χ*^2^ (1) = 4.12) than males, whilst irritability was significantly higher in males (48.4% vs 25.0%, *p* = 0.009, *χ*^2^ (1) = 6.69) than females.

In participants with FTD, the following NPS were significantly higher in males than females: delusions (21.9% vs 0.0%, *p* = 0.037, Fisher’s exact test), depression (46.9% vs 16.7%, *p* = 0.033, *χ*^2^ (1) = 4.56), apathy (68.8% vs 33.3%, *p* = 0.015, *χ*^2^ (1) = 5.86), and nighttime behaviours (65.6% vs 36.8%, *p* = 0.046, *χ*^2^ (1) = 3.99).

Lastly in participants with PD, the following NPS were significantly higher in males than females: depression (42.1% vs 22.6%, *p* = 0.048, *χ*^2^ (1) = 3.88), apathy (28.0% vs 6.5%, *p* = 0.012, *χ*^2^ (1) = 6.29), and nighttime behaviours (58.9% vs 32.3%, *p* = 0.009, *χ*^2^ (1) = 6.84). No sex differences were observed for CVD and ALS.

### Relationship amongst neuropsychiatric subsyndromes, cortical thickness regions, and WMH volumes

All cases with complete data across the five dx and variables of interest, i.e. sex, age, cortical thickness regions, and lobar WMH, were used for both the elastic net and PLSc analyses (*N* = 490). Table 4 represents the distribution of males and females per dx. For these 490 participants, the mean age = 68.67, median age = 68.78, min/max age = 40.12/87.80; the mean MoCA = 24.40, median MoCA = 25.00, min/max MoCA = 13.00/30.00.

**Table 4:** Demographics and summary for all elastic net and PLSc analyses

*N* = 490	Female	Male
ADMCI	52	65
ALS	16	23
FTD	19	32
PD	31	107
CVD	46	99

Mean age = 68.67, Median age = 68.78, Min/Max age = 40.12/87.80

Mean MoCA = 24.40, Median MoCA = 25.0, Min/Max MoCA = 13.00/30.00

*PLSc* Partial least square correlation, *AD* Alzheimer’s disease, *ALS* Amyotrophic lateral sclerosis, *CVD* Cerebrovascular disease, *FTD* Frontotemporal disease, *MCI* Mild cognitive impairment, *PD* Parkinson’s disease

### Elastic net models

The psychotic subsyndrome model produced eight lambda values that occurred greater than or equal to 5% of all resamples (i.e. >  ~ 25/500). Table 5 shows the results for the psychotic subsyndrome models. One large lambda value (1000) occurred 70/500 times which was the full sample of data that produced an intercept only model. The other seven lambda values occurred a total of 200 out of 500 times and all values were generally in the same range (0.057–0.072). All lambda values produced the same variables for selection in the full sample: age, sex (female), MoCA, left hemisphere precuneus thickness, and right hemisphere (isthmus cingulate thickness, pars-triangularis thickness, and BGT WMH). Left pars-orbitalis, left posterior cingulate, and right caudal anterior cingulate thickness did not appear across all models.

**Table 5:** Psychotic subsyndrome analyses

	1000 (70/500)	0.0692 (22/500)	0.0724 (29/500)	0.0631 (35/500)	0.0832 (23/500)	0.0575 (32/500)	0.0661 (28/500)	0.0603 (31/500)
(Intercept)	0.757	3.505	3.254	3.974	2.455	4.402	3.745	4.193
Age	0	− 0.010	− 0.009	− 0.011	− 0.007	− 0.012	− 0.010	− 0.012
Sex(female)	0	− 0.209	− 0.200	− 0.226	− 0.172	− 0.241	− 0.217	− 0.233
MoCA TOTAL	0	− 0.006	− 0.005	− 0.008	− 0.001	− 0.009	− 0.007	− 0.009
LH PARS-ORBITALIS THICKNESS	0	0.153	0.111	0.230	0.000	0.302	0.192	0.267
LH POSTERIOR CINGULATE THICKNESS	0	− 0.040	− 0.031	− 0.056	0.000	− 0.071	− 0.048	− 0.064
LH PRECUNEUS THICKNESS	0	− 0.115	− 0.091	− 0.160	− 0.019	− 0.201	− 0.138	− 0.181
RH CAUDAL ANTERIOR CINGULATE THICKNESS	0	− 0.021	− 0.008	− 0.045	− 0.000	− 0.066	− 0.033	− 0.056
RH ISTHMUS CINGULATE THICKNESS	0	− 0.090	− 0.082	− 0.104	− 0.048	− 0.118	− 0.097	− 0.111
RH PARS-TRIANGULARIS THICKNESS	0	− 0.292	− 0.258	− 0.356	− 0.155	− 0.414	− 0.325	− 0.386
RBGT WMH	0	0.116	0.104	0.129	0.075	0.144	0.121	0.137

Row names indicate variables selected, column names indicate the lambda parameter and how many times out of 500 repeats that the lambda parameter had the lowest mean square error for our repeated cross-validation. Values in the cells are coefficients from the full data sample for the corresponding selected variables (rows) under the penalisation parameter (columns)

*MoCA* Montreal Cognitive Assessment, *LH* Left hemisphere, *RBGT WMH *Right basal ganglia/thalamus white matter hyperintensities, *RH* Right hemisphere

Table 6 shows the results for the apathy subsyndrome models. The apathy subsyndrome model produced seven lambda values that occurred greater than or equal to 5% of all resamples (i.e. >  ~ 25/500). The seven lambda values occurred a total of 264 out of 500 times, and all values were generally in the same range (0.066–0.095). All lambda values produced the same variables for selection in the full sample: age, MoCA, left hemisphere (rostral middle frontal and frontal pole thickness), and right hemisphere (entorhinal, middle temporal, pars-opercularis, and pars-triangularis thickness). Note sex (female), left hemisphere (cuneus thickness, transverse temporal thickness, and frontal WMH), and right hemisphere (isthmus cingulate, transverse temporal, and medial orbitofrontal) occurred less frequently across all models.

**Table 6:** Apathy subsyndrome analyses

	0.0661 (25/500)	0.0832 (28/500)	0.0794 (50/500)	0.0955 (28/500)	0.0759 (40/500)	0.0724 (38/500)	0.0871 (55/500)
(Intercept)	8.188	7.115	7.338	6.430	7.550	7.767	6.893
AGE	− 0.017	− 0.013	− 0.014	− 0.010	− 0.014	− 0.015	− 0.012
Sex(female)	− 0.030	0.000	− 0.006	0.000	− 0.013	− 0.019	0.000
MoCA TOTAL	− 0.059	− 0.054	− 0.055	− 0.051	− 0.057	− 0.058	− 0.053
LH CUNEUS THICKNESS	− 0.042	0.000	0.000	0.000	0.000	− 0.013	0.000
LH ROSTRAL MIDDLE FRONTAL THICKNESS	− 0.494	− 0.450	− 0.460	− 0.405	− 0.470	− 0.478	− 0.439
LH FRONTALPOLE THICKNESS	− 0.097	− 0.055	− 0.065	− 0.018	− 0.074	− 0.082	− 0.043
LH TRANSVERSETEMPORAL THICKNESS	− 0.080	− 0.017	− 0.034	0.000	− 0.051	− 0.064	0.000
RH ENTORHINAL THICKNESS	− 0.146	− 0.108	− 0.116	− 0.079	− 0.124	− 0.131	− 0.099
RH ISTHMUS CINGULATE THICKNESS	− 0.055	− 0.008	− 0.018	0.000	− 0.028	− 0.037	0.000
RH MEDIAL ORBITOFRONTAL THICKNESS	− 0.040	− 0.009	− 0.016	0.000	− 0.023	− 0.029	− 0.002
RH MIDDLE TEMPORAL THICKNESS	− 0.454	− 0.475	− 0.471	− 0.491	− 0.467	− 0.464	− 0.482
RH PARS-OPERCULARIS THICKNESS	− 0.097	− 0.045	− 0.057	− 0.008	− 0.069	− 0.080	− 0.033
RH PARS-TRIANGULARIS THICKNESS	− 0.509	− 0.514	− 0.513	− 0.496	− 0.511	− 0.510	− 0.511
RH TRANSVERSETEMPORAL THICKNESS	− 0.019	0.000	0.000	0.000	0.000	− 0.001	0.000
LF WMH	− 0.023	− 0.005	− 0.009	0.000	− 0.012	− 0.016	− 0.001

Row names indicate variables selected, column names indicate the lambda parameter and how many times out of 500 repeats that the lambda parameter had the lowest mean square error for our repeated cross-validation. Values in the cells are coefficients from the full data sample for the corresponding selected variables (rows) under the penalisation parameter (columns)

*MoCA* Montreal Cognitive Assessment, *LH* Left hemisphere, *LF WMH* Left frontal white matter hyperintensities, *RH* Right hemisphere

The affective subsyndrome model produced eight lambda values that occurred greater than or equal to 5% of all resamples (i.e. >  ~ 25/500). Table 7 shows the results for the affective subsyndrome models. One large lambda value (1000) occurred 89/500 times which was the full sample of data that produced an intercept only model. The other five lambda values occurred a total of 257 out of 500 times and all values were generally in the same range (0.047–0.066). All lambda values produced the same variables for selection in the full sample: age, sex (female), sex (male), MoCA, left hemisphere (lateral occipital thickness, lateral orbitofrontal thickness, lingual thickness, pericalcarine thickness, posterior cingulate thickness, and occipital WMH), and right hemisphere (caudal anterior cingulate thickness, pars-triangularis thickness, temporal pole thickness, and BGT WMH). Also note, left superior temporal thickness, right middle temporal thickness, and right parietal WMH occurred but not in all models.

**Table 7:** Affective subsyndrome analyses

	1000 (89/500)	0.0525 (44/500)	0.0479 (24/500)	0.0631 (34/500)	0.0661 (31/500)	0.0603 (34/500)	0.0575 (48/500)	0.0549 (42/500)
(Intercept)	0.800	3.419	3.553	3.124	3.046	3.200	3.275	3.348
AGE	0.000	− 0.008	− 0.009	− 0.006	− 0.006	− 0.007	− 0.007	− 0.008
Sex(female)	0.000	− 0.121	− 0.134	− 0.090	− 0.082	− 0.098	− 0.106	− 0.113
Sex(Male)	0.000	0.009	0.011	− 0.004	0.003	0.006	0.007	0.008
MoCA TOTAL	0.000	− 0.033	− 0.034	− 0.029	− 0.027	− 0.030	− 0.031	− 0.032
LH LATERAL OCCIPITAL THICKNESS	0.000	0.614	0.657	0.519	0.492	0.544	0.566	0.591
LH LATERAL ORBITOFRONTAL THICKNESS	0.000	0.345	0.420	0.173	0.127	0.217	0.261	0.305
LH LINGUAL THICKNESS	0.000	0.424	0.442	0.384	0.375	0.393	0.405	0.414
LH PERICALCARINE THICKNESS	0.000	0.111	0.140	0.040	0.019	0.059	0.078	0.095
LH POSTERIOR CINGULATE THICKNESS	0.000	− 0.118	− 0.146	− 0.048	− 0.027	− 0.067	− 0.086	− 0.103
LH SUPERIOR TEMPORAL THICKNESS	0.000	0.000	− 0.002	0.000	0.000	0.000	0.000	0.000
RH CAUDAL ANTERIOR CINGULATE THICKNESS	0.000	− 0.279	− 0.299	− 0.233	− 0.218	− 0.246	− 0.258	− 0.269
RH MIDDLE TEMPORAL THICKNESS	0.000	− 0.019	− 0.035	0.000	0.000	0.000	0.000	− 0.009
RH PARS-TRIANGULARIS THICKNESS	0.000	− 0.726	− 0.761	− 0.633	− 0.602	− 0.622	− 0.687	− 0.707
RH TEMPORAL POLE THICKNESS	0.000	− 0.107	− 0.122	− 0.072	− 0.059	− 0.083	− 0.091	− 0.100
RH INSULA THICKNESS	0.000	− 0.040	− 0.060	0.000	0.000	− 0.004	− 0.018	− 0.028
RP WMH	0.000	− 0.002	− 0.005	0.000	0.000	0.000	0.000	0.000
LO WMH	0.000	− 0.050	− 0.055	− 0.036	− 0.032	− 0.040	− 0.044	− 0.048
RBGT WMH	0.000	0.197	0.211	0.169	0.161	0.176	0.183	0.190

Row names indicate variables selected, column names indicate the lambda parameter and how many times out of 500 repeats that the lambda parameter had the lowest mean square error for our repeated cross-validation. Values in the cells are coefficients from the full data sample for the corresponding selected variables (rows) under the penalisation parameter (columns)

*MoCA* Montreal Cognitive Assessment, *LH* Left hemisphere, *LO WMH* Left occipital white matter hyperintensities, *RBGT WMH* Right basal ganglia/thalamus white matter hyperintensities, *RH* Right hemisphere

Lastly, Table 8 shows the results for the hyperactivity subsyndrome models. The hyperactivity subsyndrome model produced seven lambda values that occurred greater than or equal to 5% of all resamples (i.e. >  ~ 25/500). The seven lambda values occurred a total of 281 out of 500 times and all values were generally in the same range (0.144–0.190). All lambda values produced the same variables for selection in the full sample: MoCA, left hemisphere (rostral anterior cingulate, superior temporal, and insula thickness), and right hemisphere (caudal anterior cingulate thickness, lateral orbitofrontal thickness, medial orbitofrontal thickness, pars-triangularis thickness, and temporal pole thickness). Left fusiform thickness and right BGT WMH were less frequent across all models.

**Table 8:** Hyperactivity subsyndrome analyses

	0.1514 (32/500)	0.1905 (30/500)	0.1738 (56/500)	0.1445 (29/500)	0.1585 (30/500)	0.1659 (54/500)	0.1819 (51/500)
(Intercept)	8.521	7.047	7.636	8.876	8.179	7.911	7.348
MoCA TOTAL	− 0.067	− 0.057	− 0.061	-0.069	− 0.065	− 0.063	− 0.059
LH FUSIFORM THICKNESS	− 0.022	0.000	0.000	− 0.034	− 0.010	− 0.001	0.000
LH ROSTRAL ANTERIOR CINGULATE THICKNESS	− 0.174	− 0.138	− 0.154	− 0.180	− 0.167	− 0.161	− 0.146
LH SUPERIOR TEMPORAL THICKNESS	− 0.372	− 0.360	− 0.369	− 0.369	− 0.373	− 0.373	− 0.364
LH INSULA THICKNESS	− 0.057	− 0.047	− 0.055	− 0.051	− 0.061	− 0.058	− 0.051
RH CAUDAL ANTERIOR CINGULATE THICKNESS	− 0.436	− 0.308	− 0.362	− 0.460	− 0.412	− 0.387	− 0.336
RH LATERAL ORBITOFRONTAL THICKNESS	− 0.158	− 0.174	− 0.169	− 0.155	− 0.163	− 0.166	− 0.171
RH MEDIAL ORBITOFRONTAL THICKNESS	− 0.244	− 0.128	− 0.177	− 0.264	− 0.222	− 0.643	− 0.153
RH PARS-TRIANGULARIS THICKNESS	− 0.698	− 0.558	− 0.616	− 0.724	− 0.670	− 0.593	− 0.587
RH TEMPORAL POLE THICKNESS	− 0.077	− 0.047	− 0.060	− 0.081	− 0.072	− 0.067	− 0.054
RBGT WMH	0.011	0.000	0.000	0.024	0.000	0.000	0.000

Row names indicate variables selected, column names indicate the lambda parameter and how many times out of 500 repeats that the lambda parameter had the lowest mean square error for our repeated cross-validation. Values in the cells are coefficients from the full data sample for the corresponding selected variables (rows) under the penalisation parameter (columns)

*MoCA* Montreal Cognitive Assessment, *LH* Left hemisphere, *RBGT WMH* Right basal ganglia/thalamus white matter hyperintensities, *RH* Right hemisphere

### PLSc

**Fig. 4:**
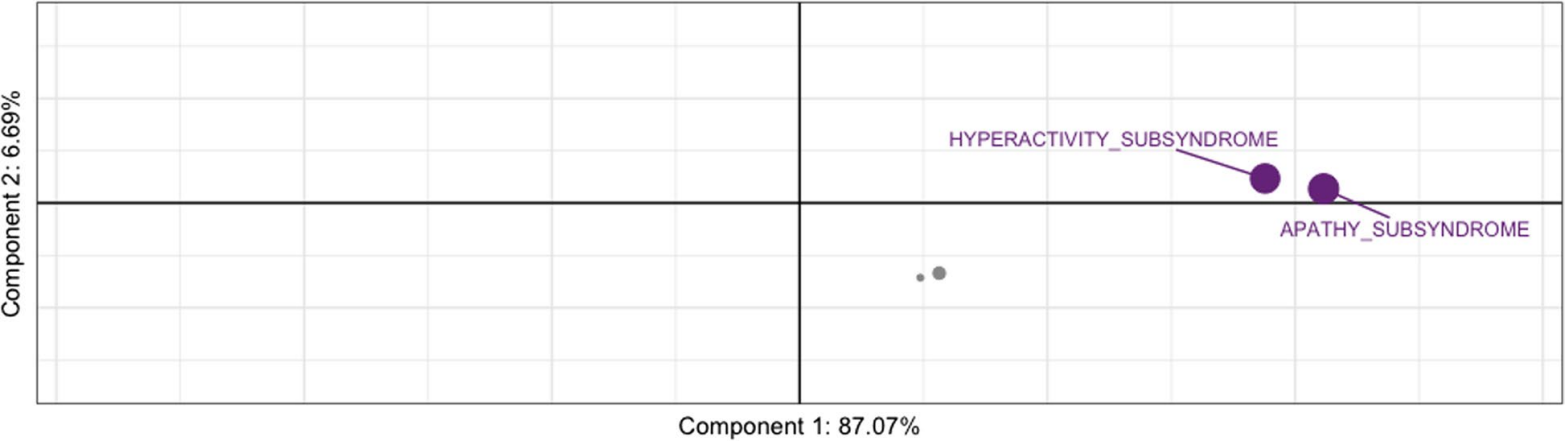
Partial least square correlation diagram for neuropsychiatric subsyndromes component scores. *Notes*: the values for all subsyndromes appear in the same direction where apathy shows the highest amount of variance on Component 1

**Fig. 5:**
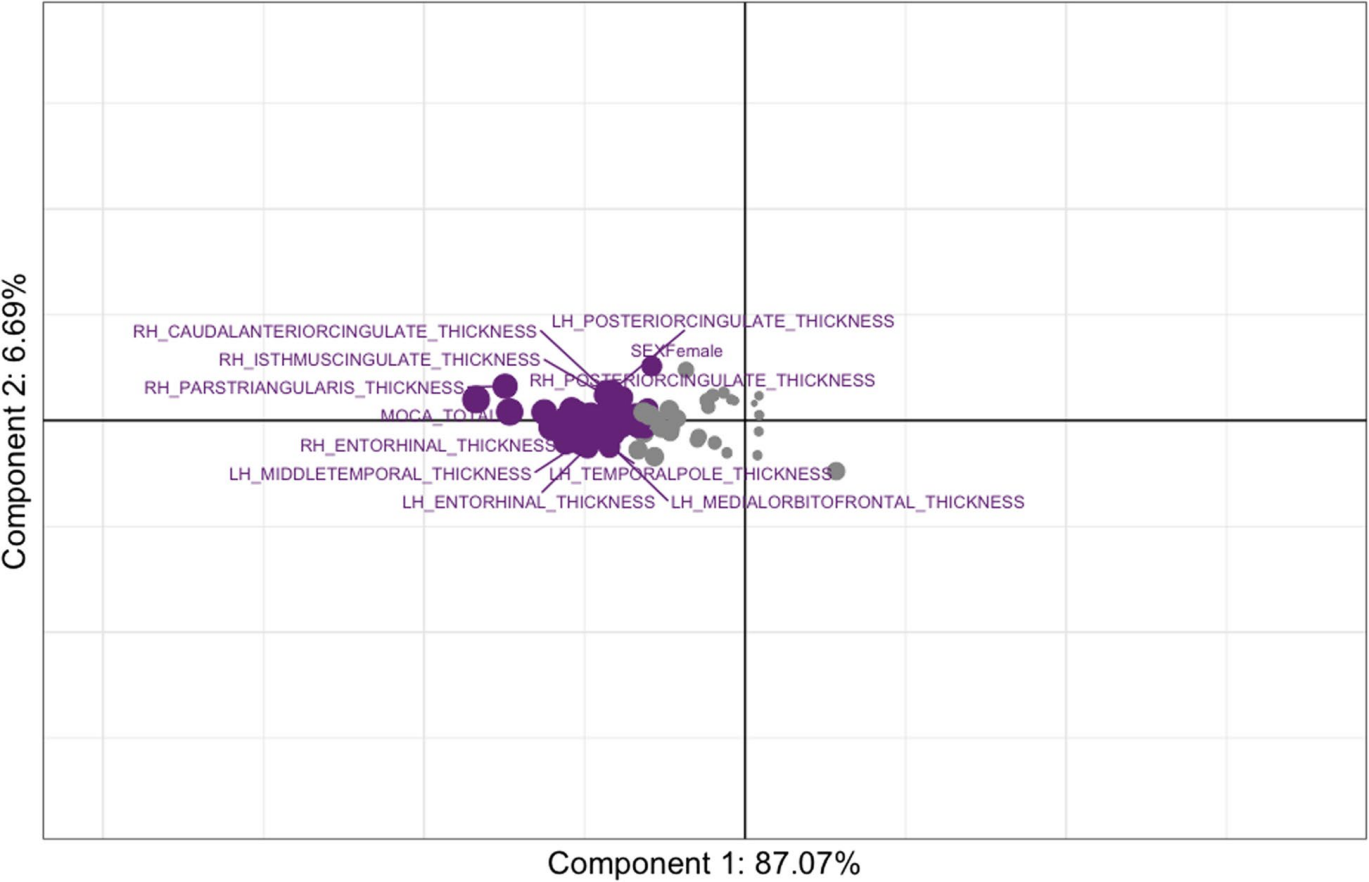
Partial least square correlation diagram for stable contributors component scores. *Notes*: the stable contributors go in the opposite direction as the neuropsychiatric subsyndromes scores, indicating a negative correlation between them. LH, left hemisphere; MoCA, Montreal Cognitive Assessment; RH, right hemisphere

**Fig. 6:**
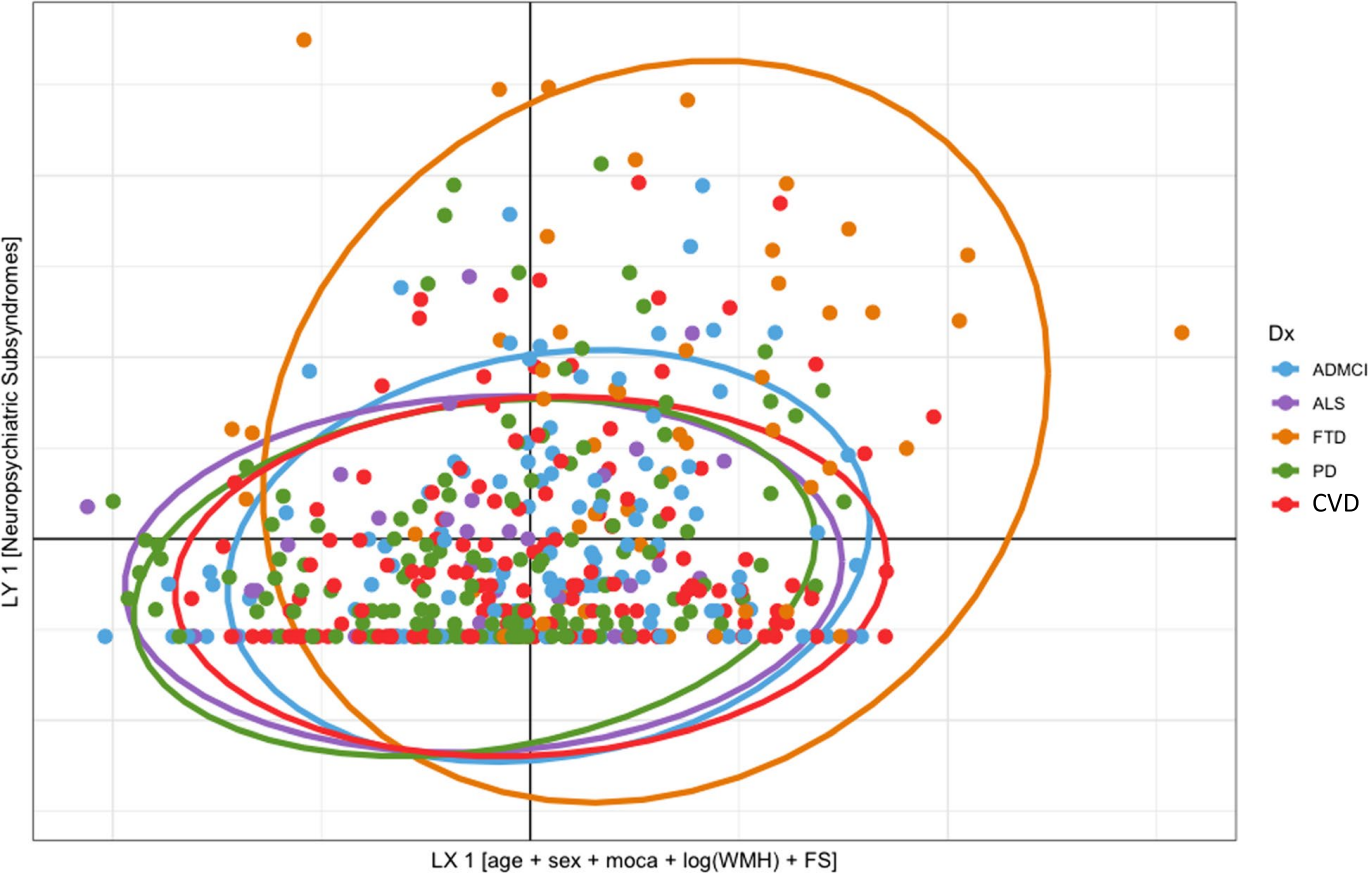
Relationship between diagnosis, neuropsychiatric subsyndromes, and contributors. *Notes*: FS, FreeSurfer cortical thickness (68 regions); WMH, lobar white matter hyperintensities (10 regions); MoCA = Montreal Cognitive Assessment; AD, Alzheimer’s disease; ALS, amyotrophic lateral sclerosis; CVD, cerebrovascular disease; FTD, frontotemporal disease; MCI, mild cognitive impairment; PD, Parkinson’s disease

The PLSc produced four components that explained: 87.07% (component 1), 6.69% (component 2), 4.09% (component 3), and 2.16% (component 4) of the variance. The *p*-values for the four components using permutation were as follows: 0.0004 (component 1), 0.1496 (component 2), 0.0236 (component 3), and 0.1092 (component 4). Although we visualised components’ 1 and 2, we only reported on component 1 due to its large variance and very low permutation *p*-value. All neuropsychiatric subsyndromes were in the same direction with apathy showing the highest amount of variance on component 1 (Fig. 4). Psychotic and affective subsyndromes were not stable contributors to component 1 (Table 9). Many predictor variables (i.e. age, sex, MoCA, and cortical thickness) were also stable contributors to component 1 (Table 10), and they go in the opposite direction as the neuropsychiatric subsyndrome scores (see Fig. 5), thus, indicating a negative correlation between dependent and predictors variables (e.g. cortical thickness). Although there were many stable predictors, it was important to highlight those that regularly appeared in the elastic net results: sex (female), MoCA, and right hemisphere pars-triangularis and anterior cingulate. They were some of the strongest contributors to component 1. Moreover, the relationship of the participants with regard to the latent variables was shown in Fig. 6 and coloured by their corresponding dx. With the exception of a few FTD participants, most of the dx were clustered together. This reflects the homogeneity in the neural correlates of NPS amongst the study participants and suggests a disease spectrum.

**Table 9:** Bootstrap ratios for the NPS subsyndromes

	**Component 1**	
Psychosis		1.202
Apathy		5.029
Affective		1.429
Hyperactivity		4.757

**Table 10:** Bootstrap ratios for all other variables (only those above magnitude of 2 are shown)

	Component 1
MoCA TOTAL	− 4.57
Sex(female)	− 2.11
LH PARS-TRIANGULARIS THICKNESS	− 3.25
LH POSTERIOR CINGULATE THICKNESS	− 2.53
LH ROSTRAL ANTERIOR CINGULATE THICKNESS	− 2.52
LH SUPERIOR FRONTAL THICKNESS	− 3.39
LH INSULA THICKNESS	− 3.44
LH PRECUNEUS THICKNESS	− 2.75
LH PARAHIPPOCAMPAL THICKNESS	− 2.68
LH FUSIFORM THICKNESS	− 2.86
LH TEMPORAL POLE THICKNESS	− 2.39
LH SUPERIOR TEMPORAL THICKNESS	− 3.83
LH MIDDLE TEMPORAL THICKNESS	− 3.19
LH PARACENTRAL THICKNESS	− 2.43
LH ROSTRAL MIDDLE FRONTAL THICKNESS	− 3.58
LH POST CENTRAL THICKNESS	− 2.24
LH PARS-OPERCULARIS THICKNESS	− 2.64
LH MEDIAL ORBITOFRONTAL THICKNESS	− 2.15
LH CAUDAL MIDDLE FRONTAL THICKNESS	− 3.09
LH FRONTAL POLE THICKNESS	− 2.40
LH TRANSVERSE TEMPORAL THICKNESS	− 3.12
LH ENTORHINAL THICKNESS	− 2.69
LH ISTHMUS CINGULATE THICKNESS	− 2.32
LH INFERIOR PARIETAL THICKNESS	− 2.02
LH INFERIOR TEMPORAL THICKNESS	− 2.69
RH CAUDAL ANTERIOR CINGULATE THICKNESS	− 2.49
RH ENTORHINAL THICKNESS	− 3.19
RH PRECUNEUS THICKNESS	− 2.77
RH INFERIOR PARIETAL THICKNESS	− 2.22
RH CAUDAL MIDDLE FRONTAL THICKNESS	− 3.18
RH MEDIAL ORBITOFRONTAL THICKNESS	− 2.51
RH LATERAL ORBITOFRONTAL THICKNESS	− 3.06
RH INFERIOR TEMPORAL THICKNESS	− 3.02
RH MIDDLE TEMPORAL THICKNESS	− 4.35
RH PARS-ORBITALIS THICKNESS	− 2.54
RH ROSTRAL ANTERIOR CINGULATE THICKNESS	− 2.03
RH SUPERIOR TEMPORAL THICKNESS	− 3.42
RH SUPRAMARGINAL THICKNESS	− 2.37
RH FRONTAL POLE THICKNESS	− 2.49
RH TEMPORAL POLE THICKNESS	− 2.99
RH PARAHIPPOCAMPAL THICKNESS	− 3.05
RH LATERAL OCCIPITAL THICKNESS	− 2.35
RH LINGUAL THICKNESS	− 2.56
RH FUSIFORM THICKNESS	− 3.32
RH ISTHMUS CINGULATE THICKNESS	− 2.89
RH PARS-OPERCULARIS THICKNESS	− 2.29
RH PARS-TRIANGULARIS THICKNESS	− 3.54
RH POSTERIOR CINGULATE THICKNESS	− 2.65
RH ROSTRAL MIDDLE FRONTAL THICKNESS	− 3.23
RH SUPERIOR FRONTAL THICKNESS	− 3.78
RH TRANSVERSE TEMPORAL THICKNESS	− 2.73
RH INSULA THICKNESS	− 2.95

*LH* Left hemisphere, *RH* Right hemisphere, *MoCA* Montreal Cognitive Assessment

## Discussion

In this study, we sought to compare NPS rates across multiple neurodegenerative and cerebrovascular diseases and determine the relative contribution of white matter lesion load and cortical thickness to NPS. The major findings included (1) NPS were common across all diseases which was consistent with the literature and (2) a smaller focal cortical thickness was significantly associated with NPS subsyndromes across all disease groups. Moreover, although there was a significant association between WMH burden and NPS subsyndromes in the univariate analyses, it was not maintained in the multivariate analyses signifying that across these diseases, focal atrophy contributed more to NPS.

We observed that participants with FTD had higher rates of agitation, anxiety, apathy, appetite changes, delusions, disinhibition, euphoria, irritability, aberrant motor behaviour, and nighttime behaviours than the other neurodegenerative or cerebrovascular groups. This is consistent with other research that showed that FTD was associated with higher rates of NPS than other neurodegenerative diseases [79–83]. This finding may be related to the early alterations in the fronto-subcortical structures seen in FTD that are responsible for various behavioural functions [79]. Such alterations are often observed in bvFTD which accounted for 40% of our FTD and are present at early stages [46, 84]. As expected, nighttime behaviours were significantly higher in PD with a trend for higher hallucinations in that group. This is consistent with the literature wherein visual hallucinations and sleep disorders are more common in PD and Dementia with Lewy Bodies (DLB) [85–87]. Psychotic symptoms are strong indicators of PD and DLB although DLB patients were excluded from our study [88–90].

The most prevalent NPS across all groups in our study was depression, which is consistent with prior studies that reported no significant difference in depressive symptoms across neurodegenerative diseases [80–82, 91]. Both anxiety and depression can be presenting symptoms of neurodegenerative disease as well as strong predictors of cognitive decline [92–97].

Sex differences were observed in our study with irritability and nighttime behaviours seen more frequently in males across the entire sample. Additionally, males with FTD or PD were more likely to experience NPS, such as delusions, apathy, and depression whilst females with AD/MCI were more likely to experience depression. Studies examining the sex/gender differences in presentation of NPS in dementia have mostly been in AD/MCI and have reported inconsistent findings [98–102]. The higher frequency of depression in females with AD/MCI is in keeping with previous studies that reported that more females suffer from affective disorders [100, 103–105]. One study found that depression was associated with a twofold greater risk of AD in females but not males [106]. The higher frequency of irritability, nighttime behaviours, delusion, and apathy in males is in keeping with some studies that also reported higher frequencies of the aforementioned NPS in males [98, 107–109], whilst contradicting others that have showed the opposite [98, 99, 105, 108]. These sex differences may be attributed to multiple factors such as disease severity across studies, the use of different NPS assessments, the genetic predisposition to AD including the interaction between sex and apoE_4_ in AD/MCI [99, 110], sex-related hormonal levels, or the use of pharmacological treatments [109]. Also, some diseases have sex differences in distribution and are associated with specific NPS, for example 50% of individuals with PD or DLB experience psychotic symptoms as compared to 30% of individuals with AD/MCI [111], but PD and DLB are more prevalent in males [112, 113]. Moreover, bvFTD appears to be more prevalent in males [114], and they have more apathy and psychotic symptoms and less empathy [71, 92, 115]. A recent study found that females with bvFTD displayed fewer NPS, particularly less apathy, sleep disturbance, and appetite changes than males, despite showing a similar amount of atrophy [116], which may support the neuroprotective role of oestrogen hormone in females [117].

A smaller cortical thickness was implicated in NPS across all groups. Although we obtained several brain regions within each NPS subsyndrome, we concentrated on those that appeared across subsyndromes and analyses such as the pars-triangularis, prefrontal, cingulate, temporal and frontal poles, and insula cortices. Apathy is a multifaceted syndrome representing deficits in cognition, emotion, and initiation [118]. It is not surprising that several studies report similar neuroanatomical correlates of apathy regardless of the underlying pathologies. Apathy is associated with changes in the fronto-striatal circuits (the dorsal anterior cingulate cortex and ventral striatum) in addition to the orbitofrontal cortex and basal ganglia [119]. In PD, the neural correlates of apathy have been structurally and functionally linked to a broad range of regions modulated by dopamine like the ventral striatum and prefrontal cortex [120–126]. Likewise in AD/MCI, Guercio et al. [20] found apathy was associated with smaller inferior temporal and increased anterior cingulate thickness in MCI whilst other studies found lower grey matter volume in the anterior cingulate, prefrontal, and subcortical areas were associated with apathy in AD [39, 127–129]. These findings in AD/MCI have been corroborated in some functional imaging studies that observed a relationship between apathy and hypometabolism in the anterior cingulate cortex and medial prefrontal cortex [130–132] in addition to being linked with increased neurofibrillary tangles in the anterior cingulate cortex [133].

The change in the anterior cingulate cortex has been implicated in apathy in FTD, ALS, and CVD. In FTD, apathy was related to atrophy in the subcortical areas in addition to anterior cingulate, and fronto-insular cortices in bvFTD [12, 134, 135]. Similar regions were associated with apathy in participants with ALS-FTD [136] and ALS without dementia [15]. Additionally, lesions in the fronto-striatal circuits has been implicated in apathy or related-disorder abulia [119, 137, 138] thus, indicating that it is a common symptom of both ischaemic and haemorrhagic strokes [138]. Moreover, functional neuroimaging has demonstrated decreased functional connectivity in the cingulo-opercular network due to dysfunction of the connecting regions [139]. Together, these results imply that the manifestation of apathy across multiple neurodegenerative and cerebrovascular diseases results from the disruption of critical and interconnected regions—mainly anterior cingulate cortex and ventral striatum—that are necessary for goal-oriented behaviours.

Psychosis was also associated with a smaller cortical thickness in fronto-cingulate and left precuneus regions. The inferior frontal and precuneus cortices have been implicated in visual hallucinations and delusions [140]. Lower grey matter volume in multiple regions including the right frontoparietal cortex were associated with delusions in AD [128] whilst decreased cortical thickness in the supramarginal gyrus was found in both AD and PD with visual hallucinations [19, 141]. Additionally, Sanchez-Castaneda et al. [142] found visual hallucinations were associated with atrophy in the precuneus and inferior frontal areas in DLB and orbitofrontal area in PD with dementia. Dysregulation amongst the frontoparietal networks has been implicated in psychosis across neurodegenerative diseases but different patterns are evident. Shine et al. [143] found an increase in connectivity between the default mode network (DMN) and ventral attention networks and a decrease in the DMN in patients with PD with visual hallucinations compared to patients without. In AD/MCI, Qian et al. [144] reported decreased connectivity between the inferior parietal lobule, superior temporal, and orbitofrontal with greater delusion severity in patients with AD compared to those without. These results suggest that disruption between top-down dorsal attention and bottom-up ventral attention and DMN processing can result in psychosis [145]. In relation to FTD, ALS, and CVD, only a few studies have explored the neural mechanism of psychosis [146, 147]. Devenney et al. [146] reported a predominant frontal and temporal pattern of atrophy extending to cerebellum and anterior thalamus across all the FTD-ALS continuum, particularly in C9orf72 carriers. Whilst Stangeland et al. [147] found the majority of post-stroke patients with psychosis had right hemisphere lesions mainly in frontoparietal and basal ganglia regions. Since these are network-based diseases, it is possible that psychosis can result from dysfunction of core neural networks that are associated with perception and beliefs in addition to interacting with other associative networks, thereby leading to disease-specific psychotic symptoms [148].

We found that increased right basal ganglia/thalamus WMH volume was associated with psychotic, affective, and hyperactivity subsyndromes whilst increased left frontal WMH volume was associated with apathy subsyndrome, albeit a lesser contributor than cortical thickness. Our results are in contrast to two recent longitudinal studies that showed that WMH contributed more to the progression of NPS subsyndromes than decreased grey matter volume in individuals with AD/MCI [39, 149]. Previous studies have demonstrated that lacunes and WMHs in the fronto-striatal circuitry were correlated with affective disorders [29, 40, 150–152], psychosis [31, 153], and reduction in goal-oriented behaviours [29, 32, 154] in neurodegenerative and cerebrovascular diseases disease. Kim et al. [32]. reported that lacunes and WMH, especially in the frontal lobe and basal ganglia and thalamic areas, were associated with depression and apathy in subcortical-vascular cognitive impairment. Similarly, a study on individuals with autosomal dominant arteriopathy with subcortical infarcts and leukoencephalopathy (CADASIL) found that basal ganglia and thalamic lesions were associated with apathy [154] whilst another reported an association between depression and frontal and temporal WMHs in community dwelling older adult [150]. In probable AD, increased frontal WMH was associated with apathy whilst increased right parietal WMH was associated with depression [29] which was supported in an autopsy-confirmed FTD and AD study [155].

The few studies that have investigated the association between WMH and NPS in PD have reported mixed results. Kraft et al. [156] found no association between global and occipital WMH with visual hallucinations in PD but two studies found that increased WMH was associated with depression and anxiety in PD [38, 157], particularly in the fronto-striatal region [38]. Another study found that baseline WMH volume was a risk factor for worsening apathy in PD [158]. These inconsistences in the localisation of WMH in relation to NPS echoes the notion that injury to multiple sites in a network may contribute to the disruption of cortico-subcortical circuits and the manifestation of NPS across many clinical constructs [159], as well as difficulty in capturing multiple NPS as a singular concept, e.g. affective.

## Limitations and strengths

The current study has several limitations and strengths. Firstly, the generalisability of our findings might be impacted due to the lack of healthy controls in our study. Secondly, we were limited from addressing the cause-effect relationships amongst WMH, cortical thickness, and NPS due to the cross-sectional nature of our study. However, as discussed above from a recent longitudinal study, WMH may contribute more to NPS progression than decreased cortico-subcortical grey matter volumes, at least in individuals with AD/MCI [39, 149]. This may suggest that at baseline, smaller cortical thickness may have the greatest influence on NPS but that WMH may impact NPS progression. Thirdly, focussing on changes in cortical thickness estimation may lead to the exclusion of the potential involvement of subcortical structures to the manifestation of NPS. Fourthly, we did not account for the use of antipsychotics, antidepressants, anticholinergics, and stimulants for treatment of NPS (which might affect symptom severity in our cohorts). Lastly, since clinical and neuroimaging parameters were used to make the diagnoses of disease categories without diagnostic biomarkers, some observed relationships in our cohorts might have been influenced by mixed pathology because it is very common and increasingly recognised in neurodegenerative diseases [160].

A main strength of our study was the inclusion of multiple neurodegenerative disease groups, especially participants with ALS, FTD, and PD. Prior research examining grey matter loss and/or WMH correlates of NPS have mostly focused on AD/MCI and CVD [31, 32, 39], occasionally on PD and FTD [155, 157], and rarely on ALS [136]. Thus, our study provides an opportunity to investigate these associations across several disease groups. Also, we were able to adjust for several factors associated with NPS in our models.

## Conclusions

Our findings demonstrate the high prevalence of NPS in neurodegenerative and cerebrovascular diseases, especially in FTD. Using both univariate and multivariate models, we showed that smaller cortical thickness and white matter lesion burden are associated with NPS subsyndromes across disease groups. In this cross-sectional study, a smaller cortical thickness was a more stable predictor than WMH in NPS across disease groups, particularly in the fronto-cingulate regions. These results underline the need for future longitudinal studies to include multiple neurodegenerative and cerebrovascular diseases when examining the interactive effects of WMH and grey matter loss on NPS. Moreover, SVD is associated with modifiable vascular risk factors, like hypertension, type 2 diabetes, and smoking that can be significantly reduced via healthy lifestyles changes. These interventions may help in managing vascular diseases that can contribute to the development of NPS in individuals with neurodegenerative and cerebrovascular diseases.

## Data Availability

The datasets presented in this article are readily available through an application process to ONDRI. For more information on the ONDRI project, please visit: http://ondri.ca/. Requests to access the datasets should be directed to http://ondri.ca/.
